# *Clinopodium* L. Taxa from the Balkans—Are There Unique Leaf Micromorphological and Phytochemical Patterns?

**DOI:** 10.3390/plants13020251

**Published:** 2024-01-16

**Authors:** Smiljana Janković, Ana Alimpić Aradski, Tanja Dodoš, Jelica Novaković, Stefan Ivanović, Ljubodrag Vujisić, Petar D. Marin, Nemanja Rajčević

**Affiliations:** 1 Facultyof Biology, University of Belgrade, Studentski trg 16, 11000 Belgrade, Serbia; alimpic.ana@bio.bg.ac.rs (A.A.A.); tanjadodos@bio.bg.ac.rs (T.D.); jelica@bio.bg.ac.rs (J.N.); pdmarin@bio.bg.ac.rs (P.D.M.); nemanja@bio.bg.ac.rs (N.R.); 2 Institute of Chemistry, Technology and Metallurgy, National Institute of the Republic of Serbia, University of Belgrade, Njegoševa 12, 11000 Belgrade, Serbia; stefan.ivanovic@ihtm.bg.ac.rs; 3 Faculty of Chemistry, University of Belgrade, Studentski trg 16, 11000 Belgrade, Serbia; ljubaw@chem.bg.ac.rs

**Keywords:** *Clinopodium*, the Balkans, micromorphological traits, *n*-alkanes, branched alkanes, cuticle, bioclimatic parameters, microadaptations

## Abstract

The concept of the genus *Clinopodium* L. has changed considerably since its first description. Most of the currently accepted species of the genus have traditionally been treated as separate genera in the group *Satureja* sensu lato: *Clinopodium* L., *Calamintha* sensu Miller or Moench, and *Acinos* sensu Miller or Moench. This study aimed to gain a better insight into the species diversity of *Clinopodium* L. from the Balkans by analyzing the taxa that have traditionally been placed in separate genera. The alkane profile and the micromorphological characteristics of the leaves are analyzed. The leaves are visualized using scanning electron microscopy, and alkanes are isolated using *n*-hexane as a solvent and analyzed using gas chromatography/mass spectrometry. The alkane profile showed the differentiation of the *Acinos*-group from the other taxa based on the dominant *n*-C31, while most of the other taxa contained *n*-C33 as the dominant alkane. The micromorphological features also showed clear differences between the previously recognized genera, especially in the capitate trichomes. The results showed that micromorphological patterns are highly variable in certain groups of the genus *Clinopodium*.

## 1. Introduction

The genus *Clinopodium* L. belongs to the family Lamiaceae Martinov, subfamily Nepetoideae (Dumort.) Luerss., tribus Mentheae Dumort., subtribe Menthinae (Dumort.) Endl) [1]. The genus comprises 186 species [2], with different habits—mostly herbaceous annual or perennial, erect to ascending shrubs, and usually inhabiting open, dry habitats and rarely closed woodland. The distribution of the genus is mainly in the New World and Eurasia, but a few members occur in northern Africa, tropical Asia, and Indomalasia [3].

The taxonomic concept of the genus has undergone great changes from its description to today. Most currently accepted *Clinopodium* species are traditionally treated as a part of the *Satureja* sensu lato group. More precisely, most members of *Clinopodium* are taxa from Saturineae and Melisineae [4] or the tribe Satureieae sensu Bentham [5]. It is difficult to summarize the position of today’s *Clinopodium* species in classification systems. Still, two prominent opinions can be found: taxonomists who supported a clear separation between the genera *Satureja* sensu Miller or Moench, *Clinopodium* L., *Calamintha* sensu Miller or Moench, and *Acinos* sensu Miller or Moench (Bentham [5], Boissier [6], Visiani [7], Šilić [8]),and those who lumped all of them under the name *Satureja* or *Clinopodium* (Briquet [9], Kuntze [10]). Based on molecular evidence, Bräuchler et al. [11,12] proposed a new name for this complex—*Clinopodium* sensu lato. The number of species is increasing by including numerous taxa from the other genera [13]. However, many authors still use the previously defined names that support a clear delimitation between genera. There is no unique scientific opinion on the concept of the genus *Clinopodium*, and additional knowledge on the biology of these taxa is needed.

The cuticle is an important interface between a plant and its above-ground environment and one of the crucial requirements for the survival of plants on land. It consists of a cutin layer and an epicuticular layer [14]. The main functions of the cuticle are to prevent water loss and protect against UV radiation, high temperature, phytopathogens, and mechanical and chemical damage [15]. The outer (epicuticular) layer comprises various compounds, including *n*-alkanes, primary and secondary alcohols, fatty acids, aldehydes, ketones, diketones, and *n*-alkyl ester [14]. The structure of the cuticle is regulated during plant development by the genotype and can change in response to environmental stresses during leaf development [16,17,18].

Alkanes are important components of epicuticular wax and are widely distributed in plants. Due to the lack of functional groups in the straight-chain molecules, *n*-alkanes are long-lived molecules easily found in diverse fossil records [19]. For the same reason, they are investigated in numerous chemotaxonomic studies [20,21,22,23]. The metastudy by Bush and McInerney [19] showed that certain *n*-alkane chain lengths predominate in certain plant groups. For example, C23 and C25 dominate in *Sphagnum* mosses, C27 and C29 in woody plants, and C31 is widespread in grasses and herbaceous plants. Since they play a significant role in plant–environment interactions, *n*-alkanes warrant more and more attention in interpreting bioclimatic changes. With this in mind, a comprehensive and systematic survey of alkane profiles among plants is needed. The importance of these investigations at the species level was recognized by Teunissen van Manen et al. [24]. The authors suggested that the limited knowledge of leaf wax *n*-alkanes at lower taxonomic levels remains unclear regarding species-specific responses’ prevalence and what characterizes them.

In a number of species, along with *n*-alkanes, the epicuticular layer also includes branched alkanes. Though these alkanes are significantly less abundant in the plant world, *iso*- and *anteiso*-alkanes (2-methyl- and 3-methyl-branched alkanes) were found in several genera from the Lamiaceae and some other families [21,25,26,27]. The function of the branched alkanes is still poorly understood. Goodwin [28] presented that methyl branching in alkanes creates steric hindrance that prevents parallel or close wax packing within the cuticle, leading to increased volatility, and that it is an adaptation to enhance scent emission. This would also lead to a poorer hydro barrier in the cuticle. When it comes to biosynthesis, the branched amino acids, valine, leucine, or isoleucine, may serve as precursors of *iso*- and *anteiso*-alkanes [29,30].

The indumentum is the hairy covering of a plant’s surface [31], which forms a protective layer for plant defense against different abiotic and biotic stresses. Various important compounds are synthesized and stored in trichomes. These structures and the compounds they contain protect the plants from pathogens, herbivores, intense evaporation, high temperatures, mechanical damage caused by wind and sand, and numerous other factors [32,33]. Although the pattern of trichome diversity is under the influence of environmental factors, the initialization and branching of trichomes are controlled by regulatory genes [34,35]. Conceivably, such a complex relationship between genetic and environmental factors in trichome development has brought about a biologically significant diversity of forms, making trichomes good taxonomic markers. The taxonomic significance of the micromorphological features of leaves, calyces, and nutlets in the Lamiaceae family and the *Satureja* complex has been widely ascertained [36,37,38]. Considering the lumping of morphologically very heterogeneous taxa from the former genera into *Clinopodium*, a great diversity of indumentum in the genus is also expected. Moreover, whether the unique, recognizable pattern of micromorphological traits exists in the genus is questionable. Wang [33] summarized that the environment, hormones, and regulatory genes determine the indumentum’s features.

This research aimed to gain a better insight into the diversity of alkanes and micromorphological characters, as well as their variability, in the investigated species and infraspecific taxa. To achieve this, taxa from the Balkan Peninsula, traditionally classified in separate genera, were selected. According to more recent studies [11,12] and the current nomenclature system [2], all previous genera are classified as a single genus: *Clinopodium*. The following taxa were used in this study: *Clinopodium acinos* (L.) Kuntze (AC), *Clinopodium alpinum* subsp. *alpinum* (L.) Kuntze (AL), *Clinopodium alpinum* subsp. *hungaricum* (Simonk.) Govaerts (ALH), *Clinopodium alpinum* subsp. *albanicum* (Kümmerle & Jáv.) Govaerts (ALA), *Clinopodium suaveolens* (Sm.) Kuntze (SU)—formerly members of the genus *Acinos* (in further text: *Acinos*-group); *Clinopodium menthifolium* (Host) Stace (ME), *Clinopodium vardarense* (Šilić) Govaerts (VA)—formerly members of the genus *Calamintha* (in further text: *Calamintha* group); *Clinopodium pulegium* (Rochel) Bräuchler (PU), *Clinopodium thymifolium* (Scop.) Kuntze (TH)—formerly members of the genus *Micromeria* (in further text: *Pseudomellisa* group); and *Clinopodium vulgare* L. (VU). This study aimed to investigate the degree of environmental influence on the alkane profile and micromorphological features in the genus *Clinopodium*. Micromorphological and chemical variability in the cuticular layer was studied in the context of microadaptations to different environmental conditions of the studied species. The correlation between the chemical composition of the epicuticular layer and the micromorphology of the leaf surface was also examined. It is the first step to understanding the possible evolutionary pathways and plasticity of the leaves’ micromorphology.

This study is a part of a larger study aimed at better understanding the distribution patterns in *Clinopodium* in the Balkans, and the presumed high taxonomic diversity in a relatively small area of the Balkans. Additionally, the study of chemotaxonomic and micromorphological patterns and variability of this genus will contribute to solving the taxonomic issues still present.

## 2. Results

### 2.1. Epicuticular Wax Composition

In the hexane extracts of leaves, normal chain and branched alkanes were detected (Table 1, for full table cf. Supplementary Table S1). All alkane extracts from the mature leaves of *Clinopodium* populations showed *n*-alkanes series from *n*-C21 to *n*-C35. The relative content of *n*-alkanes in the range from C21 to C26 was lower than 1.0% of the total leaf n-alkane content. Also, in four out of sixty-two tested samples, *n*-C36 was detected, but only in a low percentage (<1.0%). In 52 of 62 examined samples, the most abundant *n*-alkane was *n*-C33, while the remaining samples contained *n*-C31 as the quantitatively dominant component. Within four out of ten examined taxa (AC, AL, ALH, ALA), intrapopulation variability was also detected. In these populations, most samples contained either *n*-C31 or *n*-C33 as the most abundant *n*-alkane. In all tested samples, the predominance of the odd n-alkanes was observed, with *n*-C33, *n*-C31, and *n*-C29 as the main alkanes.

Series of *iso*- and *anteiso*-alkanes were detected in all 62 samples. The *Iso*- series ranged from 27 to 37, while the *anteiso*- series ranged from 29 to 36 carbon atoms. However, each individual alkane from both series was mostly present in concentrations lower than 1.0% of the total leaf alkane content. The *anteiso*-alkanes show a slight even-carbon-number predominance. Most samples exhibit a higher amount of *anteiso*-alkanes, except the two populations of VU, where *iso*-alkanes were more abundant. In most samples, *iso*-C33 and *anteiso*-C34 were the most abundant branched alkanes. The average total content of branched alkanes present per taxa varied between 5.65% (VU) and 12.95% (VA).

The average chain length (ACL) in the analyzed samples varied. The minimum chain length (31.5) was found in the *C. acinos* (AC) and *C. alpiunum* subsp. *hugaricum* (ALH) samples, while the maximum chain length (33) was found in the leaves of *C. menthifolium* (ME). This pattern corresponds to the most abundant alkane (*n*-C31 in most samples of the *Acinos*-group and *n*-C33 in *C. menthifolium*).

The relative yield of epicuticular wax (calculated based on the total FID area per g of dried plant material) varied between samples, with the lowest value in *C. alpiunum* subsp. *hugaricum* (ALH) and the highest value in *C. menthifolium* (ME) from Zlot. Most samples contained a relative wax content between 15 and 40% percent of the highest yield (ME1).

#### Statistical Analysis of Alkane Profile

The one-way variance analysis (ANOVA) was performed with all ten taxa, considering the leaf wax content of all identified *n*-, *iso*-, and *anteiso*-alkanes. Significant differences between the taxa were found for most of the analyzed alkanes. However, the more conservative pairwise Tukey test did not show one compound that could differentiate all of the taxa (Supplementary Table S1). Based on the ACL, a general trend of separation between aromatic and non-aromatic species was found, except for *C. pulegium* (PU), which was not significantly different from the other taxa.

The first two principal axes of the Principal Component Analysis (PCA) explained 89.8% of the total variance. Two components, *n*-C31 and *n*-C33, showed the most significant influence, forming two main groups. The PCA confirmed the separation of the *Acinos*-group and *C. pulegium* (PU) from the other taxa based on a higher *n*-C31 abundance. All other populations were grouped based on a high amount of *n*-C33, though separated on the second axis. The highest inter- and intrapopulation variability was found in *C. menthifolium* (ME).

**Figure 1:**
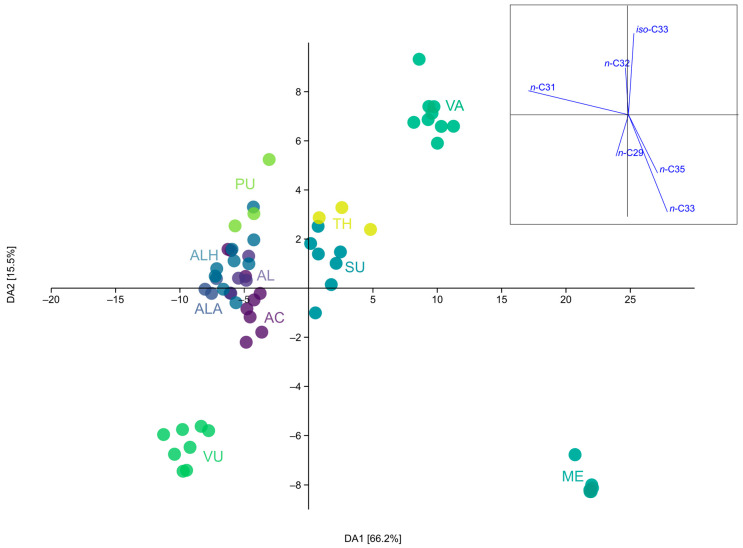
DA Scatter plot. AC—*C. acinos*; AL—*C. alpinum* subsp. *alpinum*; ALA—*C. alpinum* subsp. *albanicum*; ALH—*C. alpinum* subsp. *hungaricum*; ME—*C. menthifolium*; PU—*C. pulegium*; TH—*C. thymifolium*; SU—*C. suaveolens*; VA—*C. vardarense*; VU—*C. vulgare*.

Discriminant analysis (DA) with taxa as groups showed a clear separation into five clusters (Figure 1). Three components were most significant for separating the taxa in the DA—*n*-C33, *n*-C31, and *iso*-C33—with a significant contribution by three more (*n*-C29, *n*-C32, and *n*-C35). The ME, VA (*Calamintha*-group), and *C. vulgare* (VU) separated strongly from each other and other taxa. All six samples of *C. menthifolium* (ME) (three populations tested) form a group based on the dominance of *n*-C33 and a high abundance of *n*-C35. Only this taxon showed a specific alkane profile, which stood out from the other samples, while *C. vulgare* (VU) is separated from the others by higher concentrations of *n*-C29. A clear separation was also seen in *C. vardarense* (VA), based on the significant presence of *iso*-C33. Taxa from the *Acinos*-group mainly form the fourth group, which clustered together with *C. pulegium* (PU), primarily due to high *n*-C31 concentrations and a considerable amount of branched alkanes. The populations of *C. thymifolium* (TH) and *C. suaveolens* (SU) stand out slightly from this group due to a higher concentration of *n*-C33 and *iso*-alkanes. Interestingly, these two taxa form a kind of transition group between aromatic and non-aromatic taxa.

**Figure 2:**
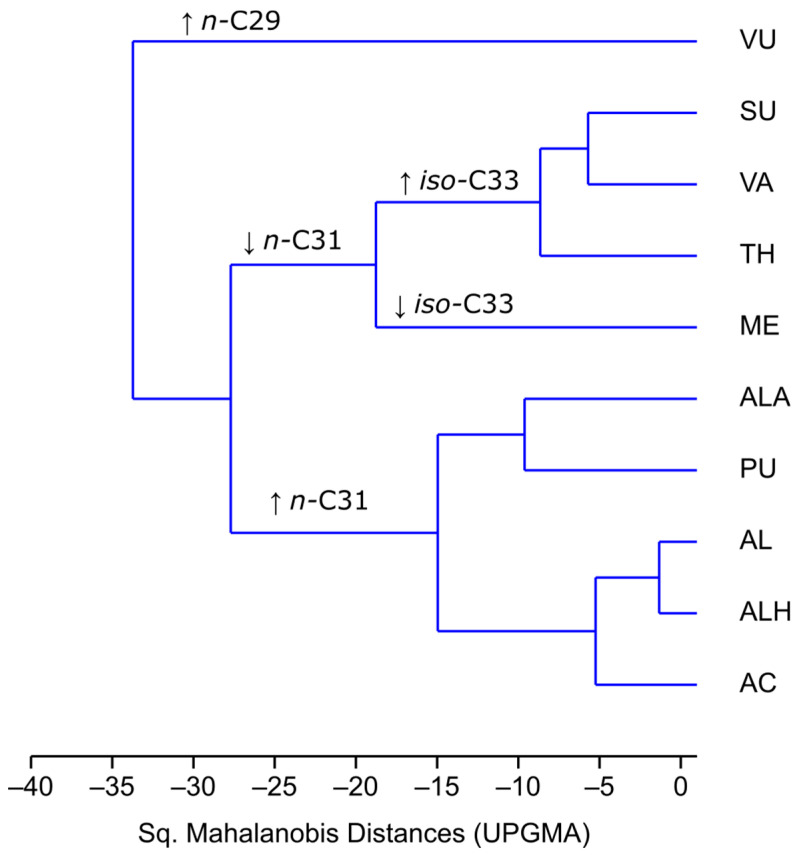
HCA cladogram (Sq. Mahalanobis distance, UPGMA) based on leaf alkane profiles.AC—*C. acinos*; AL—*C. alpinum* subsp. *alpinum*; ALA—*C. alpinum* subsp. *albanicum*; ALH—*C. alpinum* subsp. *hungaricum*; ME—*C. menthifolium*; PU—*C. pulegium*; TH—*C. thymifolium*; SU—*C. suaveolens*; VA—*C. vardarense*; VU—*C. vulgare*. Arrows pointing upwards indicate that a certain alkane is present in high concentrations and arrows pointing downwards indicate that a certain alkane is present in low concentrations.

Hierarchical cluster analysis (HCA) was performed considering only the alkanes that were significant for separation in the DA test. The HCA (Figure 2) showed a separation of the three groups. Taxon *C. vulgare* is separated from all other taxa. Linked with DA analysis, *n*-C29 is responsible for this dissimilarity. The following two separated groups are non-aromatic taxa, including all taxa of the *Acinos*-group and the group of aromatic taxa (*Pseudomellisa* and *Calamintha* groups). The separation between these two groups is due to higher concentrations of *n*-C33, *n*-C35, and *iso*-C33 in aromatic taxa and higher concentrations of *n*-C31 in non-aromatic taxa. Interestingly, *C. pulegium* (PU) is categorized with the *Acinos*-group, although it is a highly aromatic plant. *C. suaveolens* (SU) is grouped with other aromatic species, separately from the morphologically closest *Acinos*-group.

### 2.2. Micromorphological Features

In all populations analyzed, the cuticle was smooth, with no crystals or other wax structures observed. The thickness of the cuticle varied between the taxa but was not species-specific. The leaves of all species studied bore non-glandular and glandular (peltate and capitate) trichomes. The greatest diversity was observed in the morphology of capitate trichomes; there was less diversity in non-glandular trichomes, while peltate trichomes showed the most uniform morphological pattern. The occurrence of all detected trichome types among the studied taxa is presented in Table 2. The descriptions of each trichome type are given in Supplementary Table S2). The micrographs of all 24 analyzed populations are presented in Supplementary Figures S1 and S2.

**Figure 3:**
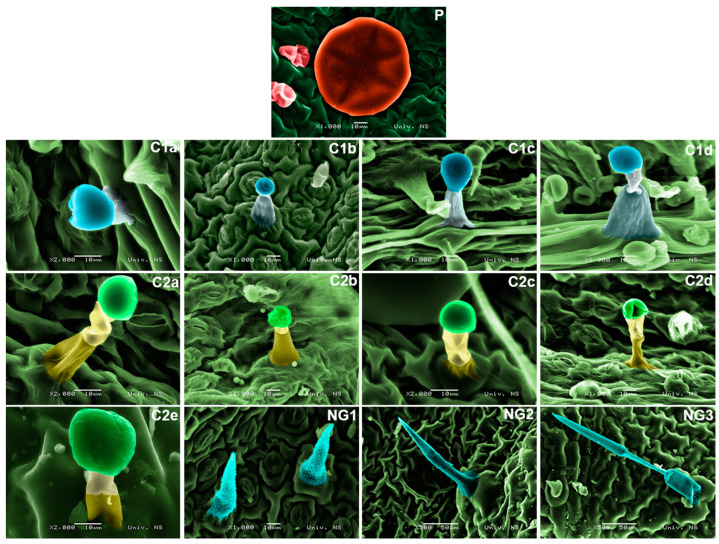
SEM micrographs of all trichome types observed in examined *Clinopodium* species. P—peltate trichomes; C1a–C2e—different subtypes of capitate trichomes; NG1–NG3—non-glandular trichomes.

**Figure 4:**
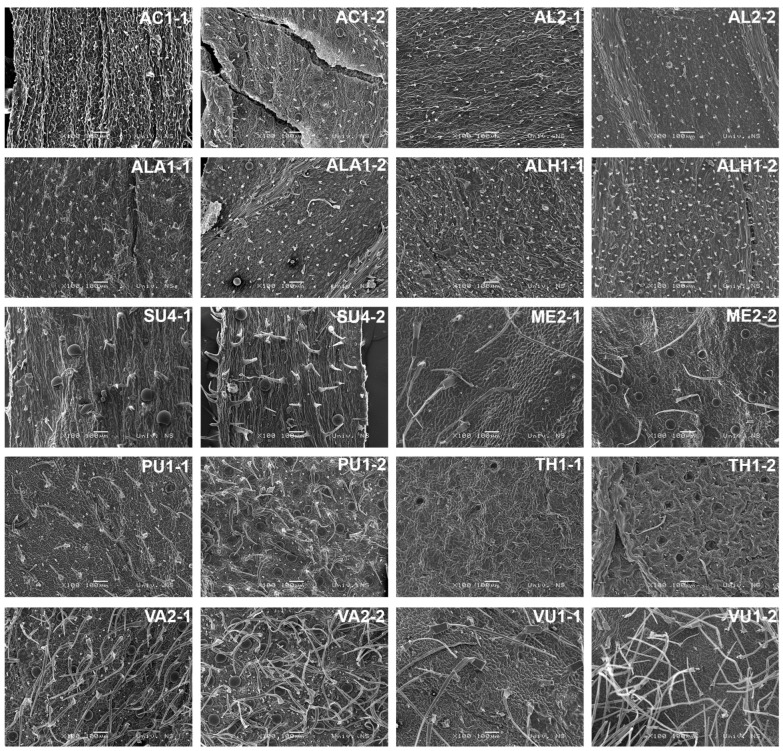
SEM micrographs of all observed *Clinopodium* taxa. For population details, cf. Table 3; adaxial (1) and abaxial leaf side (2). AC1—*C. acinos*; AL2—*C. alpinum* subsp. *alpinum*; ALA1—*C. alpinum* subsp. *albanicum*; ALH1—*C. alpinum subsp. hungaricum*; SU4—*C. suaveolens*; ME2—*C. menthifolium*; PU1—*C. pulegium*; TH1—*C. thymifolium*; VA2—*C. vardarense*; VU1—*C. vulgare*.

The peltate trichomes observed were characteristic for the Lamiaceae family (Figure 3). They consisted of a short stalk deeply embedded in the epidermis and a broad, multicellular head with a large subcuticular space. The variation in this trichome type is evident in terms of the distribution on the leaf side, trichome diameter, and density. All the taxa studied possessed peltate trichomes. Scanning electron microscopy (SEM) micrographs of all examined taxa are given in Figure 4. In most populations, peltate trichomes were present only on the abaxial leaf side, while almost all aromatic taxa, excluding *C. menhifolium* (ME), possessed peltate trichomes on both sides. The diameter of the peltate trichomes varied between 50 (in the *Acinos*-group) and 100 μm (in the *Calamintha*-group). The density of peltate trichomes varied greatly between the studied populations, with the highest density in the taxa of the *Pseudomellisa*- and *Calamintha*-groups (aromatic taxa) which clearly differ from the *Acinos*-group and *C. vulgare* (VU) with the lowest density (Table 2).

The capitate trichomes observed could be classified in two main subtypes: those with a unicellular stalk (C1 subtype) and those with a bicellular stalk (C2 subtype). Four subtypes could be distinguished in the C1 type and as many as five subtypes in the C2 type, based on shape and diameter (Figure 3).The subtype observed in all studied populations was C1a, on both the adaxial and abaxial leaf sides. This is the only subtype that is consistently present in each population. All the other subtypes of capitate trichomes are rarer and sometimes specific to a population or subspecies. The greatest diversity of all detected capitate trichomes was observed in the *Acinos*-group, especially in *C. alpinum* subsp. *hungaricum* (ALH), where each population had at least three to six different subtypes. All taxa of the *Acinos*-group showed interpopulation (infraspecific) variability in the structure of the indumentum and the presence of different capitate trichomes. In the *Pseudomellisa*-group and *Calamintha*-group, clear differences were observed between taxa in the presence of capitate trichomes.

The non-glandular trichomes were unbranched, papillose, and tapered towards the top in all examined populations. The variability in these trichomes is mainly seen in the length and number of cells and their distribution and density on the leaves. Therefore, the three main subtypes of these trichomes are distinguished, noted as NG1, NG2, and NG3 (Figure 3, Table 2). Most of the tested populations possessed all three types of non-glandular trichomes. The densest indumentum regarding the non-glandular trichomes was present in three taxa: *C. vulgare* (VU), *C. pulegium* (PU),and *C. vardarense*(VA). In these species, almost the entire surface was covered with non-glandular trichomes, and the NG3 subtype was predominant. All other taxa had less dense indumenta in terms of non-glandular trichomes.

#### Statistical Analysis of Leaves’ Indumentums

**Figure 5:**
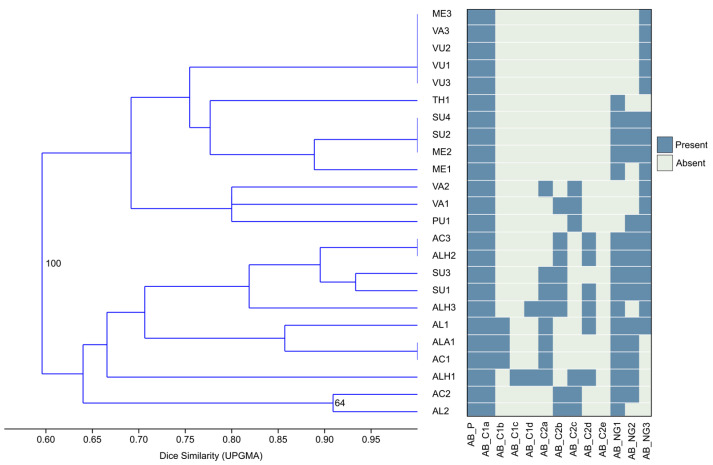
Hierarchical cluster analysis (Dice similarity, UPGMA) of abaxial leaf side trichomes’ presence/absence matrix. Bootstrap values higher than 60 are shown in nodes.

Statistical analysis of all 24 populations showed that the abaxial (AB) was more informative than the adaxial leaf side (AD). This is due to the higher number of trichome types present on the abaxial side and too much variability on the adaxial side, with a frequent absence of some trichome types. The dendrogram obtained through hierarchical cluster analysis (HCA) of the presence/absence matrix of trichomes present on the abaxial leaf side showed the separation of the two main groups: the *Acinos*-group and all other taxa (Figure 5). Interestingly, *C. suaveolens* (SU) was separated into two groups—the two populations from Serbia clustered with the *Acinos*-group, and the North Macedonian populations grouped with the other taxa.

### 2.3. Environmental Parameters

To determine if the climate might have contributed to the alkane profiles or indument characteristics, bioclimatic parameters were also analyzed. The PCA of 19 bioclimatic parameters showed a clear separation of the two bioclimatic groups, corresponding to Koppen–Geiger climate types, but also two subgroups within continental populations separating northeastern and southwestern localities (Supplementary Figure S3).

In order to determine the potential of alkanes as chemotaxonomic markers in this group and their dependence on environmental conditions, sympatric populations were sampled at four localities (cf. Table 3). PCA and MANOVA showed a statistically significant separation of epicuticular leaf profile in each locality between sympatric taxa. The collected individuals from sympatric populations were completely interspersed, sometimes growing only a few centimeters apart, and mostly completely exposed in their open habitats, meaning they were exposed to almost the same environmental factors (Supplementary Figure S4), suggesting strong genetic determination of the alkane profiles and their potential significance as chemotaxonomic markers. However, considering the samples were pooled with several individuals, we cannot completely exclude the influence of the finely nuanced microhabitats present in each locality and each individual response to them.

The pH values of soil were also measured. pH values ranged from acidic (pH = 6.1, Rogozna mountain) to mildly alkaline (pH = 7.8, Raec), but the same species could be found across the range (Supplementary Figure S5). The highest soil pH variability was found for *C. vulgare* (pH 6.1–7.4). Other non-aromatic taxa (*Acinos*-group without SU) grew on acidic-to-neutral pH soils, while aromatic taxa were more common on the neutral-to-alkaline soils (*Pseudomellisa*-group and *Calamintha*-group, with SU).

### 2.4. Correlations of the Data

The Pearson linear correlation test was used to test the possible relationship between the profile of the alkanes and the environmental parameters. Most of the alkanes did not show a simple linear correlation. The relative wax yield did not correlate with taxon or environmental parameters. Additionally, when tested individually (each species separately), several correlations were found between alkanes and environmental parameters. However, there is no apparent pattern in these correlations, suggesting that these correlations might be an artefact, due to the high variability in characters and low number of different localities, or a more complex relationship. The only exception is *C. suaveolens* (SU), where *n*-alkanes shorter than C29 were highly negatively correlated with temperature isothermality (i.e., overall annual variability in temperature), while longer ones showed a moderate positive correlation.

#### Correlation between Micromorphological Parameters

To understand the possible patterns of leaf microadaptations and the connection between the cuticle and trichome development, the Pearson linear correlation test was used. The frequency of capitate trichomes on both the abaxial and adaxial sides and peltate trichomes on the adaxial side is negatively correlated with the average content of *n*-alkanes (R = −0.50, R = −0.58, R = −0.43, respectively; *p* < 0.05). On the other hand, the peltate trichomes are positively correlated with the average content of branched alkanes (R = 0.52; *p* < 0.05). A positive correlation is also observed between the thickness of the cuticle and the frequency of peltate trichomes on the abaxial side (R = 0.41; *p* < 0.05). Additionally, the correlations between each trichome type and cuticle and alkane profile are determined. A negative correlation is found between the NG1 subtype on the abaxial side and cuticular thickness (R = −0.45; *p* < 0.05). A positive correlation is found between the C2c subtype only on the abaxial side and the cuticle thickness and branched alkanes (R = 0.52; *p* < 0.05), while the same trichome subtype is negatively correlated with the percentage of *n*-alkanes (R = −0.52; *p* < 0.05).

## 3. Discussion

### 3.1. Normal Chain and Branched Alkanes

Maffei [26] investigated if *n*-alkanes and *iso*-alkanes could be chemotaxonomic characters in the Lamiaceae family, testing the numerous taxa from different genera. He showed that the most abundant *n*-alkane is not unique to the family, and there is great diversity at the intergeneric level. According to this research, one of the *n*-alkanes in the range from *n*-C29 to *n*-C33 could be recognized as a characteristic marker for a specific genus in Lamiaceae. In the former genus, *Calamintha*, the dominant *n*-alkane was C33, which is in accordance with the present results. Both taxa from the *Calamintha*-group (ME and VA) in the present study contained the dominant C33. Also, it is noted that in the genus *Teucrium*, *n*-C33 was also the dominant alkane, the same as most taxa from the genus *Clinopodium* investigated in the current study. This could be interesting since these genera are in different subfamilies but oftentimes share the same habitat. A study by Huang et al. [27] showed that leaves of *Clinopodium chinense* from three different localities always contain *n*-C33 as the most abundant alkane, which agrees with our results obtained for the *Pseudomellisa*-group and *Calamintha*-group, and *C. vulgare*. Dodoš et al. [22,39] investigated three subspecies of *Satureja montana* and *Satureja subspicata* and concluded that the dominant alkanes were *n*-C29 and *n*-C31. Interestingly, this alkane profile is most similar to the *Acinos*-group. On the other hand, the species with a very narrow distribution range, *S. kitaibelli*, showed differentiation between populations based on the abundance of C29 and C33, although the dominant alkane was C31 [40]. A similar interpopulation variability is found in *C. menhifolium* (ME), while other taxa did not show as much variability between populations. These results suggest that species of the same genus do not show the same pattern of interpopulation variability. This is likely due to the specific chorology of each species and possible genetic isolation, as suggested by the authors in [40]. In addition, Dodoš et al. [40] showed that the correlations between bioclimatic parameters and n-alkanes are significantly correlated in the genus *Satureja*. In the mentioned study, shorter n-alkanes showed a negative correlation with altitude, while longer-chain alkanes showed a positive correlation. We did not find this correlation in the taxa of the genus *Clinopodium*. We consider this to be a completely different adaptive response to the environment and a different biology of the taxa, although *Satureja* and *Clinopodium* are quite related. Reddy et al. [21] showed that four investigated *Micromeria* species show a high carbon preference index (CPI) with dominant *n*-C29 or *n*-C31. However, in their investigation of *C. thymifolium* from Serbia (=*M. thymifolia*), *n*-C31 was a dominant *n*-alkane, which does not correspond to our results, suggesting possible interpopulation variability in the alkane profile of *C. thymifolium*.

Among the first reports of *iso*- and *anteiso*-alkanes in plant leaves were the studies on *Nicotiana tabacum* [41,42]. These studies showed that the compounds with odd numbers of carbon atoms were predominant for the series of *iso*-alkanes, similar to *n*-alkanes. Huang et al. [27] suggested that a generally strong even/odd predominance of *iso*- and *anteiso*-alkanes in plant samples is attributed to different biosynthetic pathways for long-chain *iso*- and *anteiso*-alkanes. The start molecule for *iso*-alkane biosynthesis is 2-methyl propanol (C4)-CoA “starter”, while *anteiso*-alkanes begin biosynthesis with 2-methyl butanol (C5)-CoA “starter”, and are then elongated by C2 units, followed by decarboxylation, resulting in an even-numbered dominance. However, Busta and Jetter [43] indicated that it is unclear whether preference for *iso* branching is established by selecting a certain amino acid or by discriminating between *iso* and *anteiso* products in downstream metabolic steps. In all examined taxa of our study, the *anteiso*-alkanes showed even predominance, while more abundant *iso*-alkanes were odd-numbered. This is in accordance with Reddy et al. [21] and Huang et al. [27]. Reddy et al. [21] found a series of branched alkanes in *Micromeria,* which has the same distribution pattern as the genus *Clinopodium*. However, *C. thymifolium* (=*Micromeria thymifolia* in the mentioned study) shows the domination of *anteiso*-C34, while in the present study, *anteiso*-C32 was more abundant. According to present results and the literature data, *anteiso*-alkane distribution constantly exhibits even predominance in *Clinopodium*, but the most abundant branched alkane varied between populations. Maffei [26] suggested that *iso*-C32 can be used as a chemotaxonomic marker to discriminate Nepetoideae from Lamioideae and Teucrioideae. Yet, all taxa from our study possessed continuously low concentrations of *iso*-C32 even though they belong to the subfamily Nepetoideae. Additionally, these compounds were not reported for *Satureja* [22,39,40].

### 3.2. Micromorphological Features

Peltate trichomes are morphologically quite similar in the Lamiaceae family [44], although the number of head cells varies [45,46]. Our study also showed no significant differences between the taxa of the genus *Clinopodium* in terms of peltate trichome morphological features. Yet, the distribution of the peltate trichomes could be an additional character for taxa delimitation. A great variability between and within populations was found for almost all taxa, especially in the *Calamintha*-group. Nevertheless, the distribution of peltate trichomes could be a useful characteristic at the interspecific level in the mentioned *Calamintha*-group. In all three studied *C. menthifolium* (ME) populations, the peltate trichomes were present only on the abaxial leaf side, while in the *C. vardarense* (VA) populations, the peltate trichomes were present on both leaf sides. The same results for both taxa are reported by Husain et al. [47]. Most members of the *Acinos*-group had peltate trichomes only on the abaxial leaf side, except for *C. suaveolens* (SU),which is the only aromatic species from the *Acinos*-group. The populations of SU from Serbia had peltate trichomes only on the abaxial side, while in North Macedonian populations, they were present on both sides. The population from Turkey [48] also possessed peltate trichomes on both leaf sides. According to our research, the interpopulation morphological variability found in leaf shape [8] could also be seen at the micromorphological level in this species.

Capitate trichomes show a lot of intrageneric variation in the Lamiaceae family [49], which is consistent with our findings. The greatest variability among the analyzed groups can be observed in capitate trichomes. However, it is important to define which capitate trichomes contribute the most to variability. The trichome type with the shortest stalk, referred to as the C1a subtype in our study, is widespread in the family Lamiaceae, especially in the subfamily Nepetoideae [38,50]. The trichomes very similar to C1a are found in the subfamily Lamioideae [51]. Most authors interpret this type of trichome in the same way as those of the subfamily Nepetoideae. However, it seems that their morphology is quite different—the head is regularly round and not elliptical in the Lamioideae subfamily. In our research, the C1a subtype is constantly present on both leaf sides in every sample. This result, with emphasis on previously mentioned studies, shows that small capitate trichomes (in this study named C1a) are characteristic of the subfamily Nepetoideae, and we found this type does not show potential for taxa delimitation in the studied group.

In addition, there are a few cases where capitate trichomes are a useful delimiting characteristic in identifying morphologically complex taxa in heterogeneous groups. For example, in *C. menthifolium* (ME), only the C1a subtype of capitate trichomes was detected, whereas *C. vardarense* (VA) had three additional subtypes. Interestingly, VA showed variability between populations, but always had at least two subtypes of capitate trichomes. In the *Pseudomellisa*-group, in addition to subtype C1a, *C. pulegium* (PU) also had C2c, whereas in *C. thymifolium* (TH) only C1a was present. The greatest diversity of capitate trichomes was detected in the *Acinos*-group. All taxa from the *Acinos*-group had at least three subtypes of capitate trichomes, but all taxa showed interpopulation variability. This group had the most complex indumentum. On the other hand, *C. vulgare* (VU) showed no interpopulation variability in the structure of the indumentum and the greatest similarity to *C. menthifolium* (ME), except peltate trichomes. It is worth mentioning that *C. menthifolium* shows no variability between indumentum structures, despite the great morphological differences.

There is great diversity in the density and variability of non-glandular trichomes between taxa and populations (Table 2). For example, in all three populations of *C. vardarense* (VA), only NG3-subtype trichomes were detected, in contrast to *C. menthifolium* (ME), which always has NG1-, NG2-, and NG3-subtype trichomes, suggesting that non-glandular trichomes may also be an additional feature for species delimitation in this complex group.

Since the morphologically heterogeneous genera were transferred to *Clinopodium*, significant differences between the taxa groups were to be expected. However, the study by Kremer et al. [50] supported the transfer of species from the section *Pseudomellisa* (broad-leaved species of the former genus *Micromeria*) into *Clinopodium*. The authors argued the transfer with the fact that the section *Pseudomellisa* possesses an additional two types of capitate trichomes which are not noticed in typical, narrow-leaved *Micromeria* species. Still, these types of trichomes, which are considered a taxonomical characteristic, are mainly present only in floral regions. The present analysis of leaves from the section *Pseudomellisa* (i.e., *Pseudomellisa*-group) showed results in agreement with the studies by Kremer [50] and Stojičić [52], with the most abundant C1a subtype of trichomes on the leaves. The population of *C. pulegium* from Podvis, included in the present research, contained one additional type of capitate trichome not noticed in the Kremer study (Međeđa population) [50]. This interpopulation variability is probably present due to the complex chorology of this species.

According to our results, a great diversity of indumentum patterns can be observed in the genus. The *Acinos*-group had nine subtypes, the *Calamintha*-group had four subtypes, *Pseudomellisa* had two subtypes, and *C. vulgare* (VU) had only one subtype of capitate trichomes. Furthermore, according to the statistics, two main groups can be distinguished when all trichome types are compared. These are the *Acinos*-group (except SU3 and SU4), which is primarily separated by the highest number of capitate trichomes, and another group comprising the *Calamintha*-group, *Pseudomellisa*-group, and *C. vulgare*. It is questionable whether this great variability in the structure of the indumentum between the groups supports the currently accepted concept of a single genus. Including more leaves per population for SEM observation would allow higher resolution at the intrapopulation level and perhaps reveal additional information about micromorphological patterns.

### 3.3. Correlations

#### 3.3.1. Influence of the Environment

It is well known that the final morphology of the leaf, including the indumentum and cuticle development, presents a complex adaptive response to the environment. Recent studies [53,54] show that the synthesis of alkanes from very-long-chain fatty acids (VLC) is under the control of the SlCER gene group. In addition, this study showed that the alkane profile and micromorphological features did not correlate with the environmental parameters. When the leaf alkane profiles of individual species were analyzed for correlation with bioclimatic and other environmental data, no particular pattern of variability was found, so the alkane profile could not be linked to differences in any of the environmental parameters, and no proof of microadaptations in these characteristics could be established. The same holds for most micromorphological characteristics. The only exception was *C. suaveolens* (SU), which showed different patterns of glandular trichome distribution and *n*-alkane profile (but not branched alkanes) based on the significantly different climates in localities in Serbia and North Macedonia. However, the overall absence of proof of microadaptations could also be due to the relatively low number of localities and their rather similar bioclimatic characteristics. Nevertheless, the present data imply that both phytochemical and micromorphological characters are under strong genetic control and could be reliable in the taxonomy of this group.

#### 3.3.2. Correlation of the Micromorphological Features and Alkane Profile

In the study by Huang et al. [27], a comparative analysis between different plant organs showed that flowers contain a significantly higher proportion of branched alkanes than leaves and stems. This localization is probably related to the regulation of scent emission. Regardless, the function of the branched alkanes in the leaves is still poorly understood. However, the correlations found in this study could explain the possible function of these compounds. According to our results, the more glandular trichomes (both capitate and peltate) present on the leaves, the greater the ratio of *iso*- and *anteiso*- alkanes in the epicuticular wax profile. This result agrees with the explanations of Goodwin [28] and Huang et al. [27]. Of the species analyzed in our study, the most aromatic species, *C. vardarense* (VA), had the highest average content of branched alkanes in the leaves. So, the *iso*- and *anteiso-alkanes* could play the same role in the leaves as in the flowers—they enhanced the emission of volatiles.

## 4. Materials and Methods

### 4.1. Plant Material

All populations were collected during the flowering phase in 2020 and 2021 in the territories of Serbia and North Macedonia. Leaves were separated from the sampled populations, air-dried, and stored in paper bags at room temperature until phytochemical and micromorphological analysis. The voucher specimens were deposited in the BEOU (Herbarium of the University of Belgrade, Faculty of Biology and Botanical Garden Jevremovac). Details of the collected material are given in Table 3.

### 4.2. Extraction of Leaf Alkanes

Each sample was prepared by grouping the whole leaves from several plants from the population to obtain a sufficient mass for the extraction (usually 2–5 assembled plants per sample). Most of the tested populations contained three pooled samples to better represent population variability, with a few exceptions where the populations were very small-numbered or individuals had tiny and small leaves. The main setbacks of this approach are that it reduces variability due to averaging, and individual phenotypic responses to the environment are downsized.

The total amount of epicuticular waxes was extracted by immersing the aliquots from 0.2 to 0.5 g in an appropriate volume of *n*-hexane for 60 s. Then, the solvent was evaporated under a vacuum. All samples were chromatographed on a small-scale column using a Pasteur pipette filled with activated silica gel. The wax samples were obtained via elution with 4 mL of hexane, concentrated to 0.2 mL, and stored at 4 °C in amber glass vials until GC-FID and GC/MS analyses.

### 4.3. The GC-FID and GC/MS Analysis

Chemical analyses were performed with an Agilent 7890A apparatus equipped with an auto-injection system (Agilent 7683B Series; Agilent, Santa Clara, CA, USA), an inert 5975C XL EI/CI mass-selective detector (MSD), and a flame ionization detector (FID) connected with cap. flow technology 2-way splitter with make-up and an HP-5 MS cap. column (30 m, 0.25 mm i.d., film thickness 0.25 mm). The GC oven temperature was programmed from 60 to 300 °C at a 3 °C/min rate and held for 10 min. Helium was the carrier gas at 16.255 psi (constant pressure mode). An auto-injection system (Agilent 7683B Series Injector) was employed to inject 1 µL of the sample. The sample was analyzed in the splitless mode. The injector temperature was 250 °C and the detector temperature 300 °C. MS data were acquired in the EI mode with scan range 40–600 *m*/*z*, source temperature 230 °C, and quadrupole temperature 150 °C; the solvent delay was 3 min. A library search and mass spectral deconvolution and extraction were performed using NIST AMDIS (Automated Mass Spectral Deconvolution and Identification System) software version 2.64.113.71, using retention index (RI) calibration data analysis parameters with strong level and 10% penalty for compounds without an RI. The search was performed against our own library, containing 4972 spectra. The relative abundance of the *n*-alkanes was calculated from the signal intensities of the homologs in the GC/FID traces, and normalized to the highest abundance.

### 4.4. The Average Chain Length and Carbon Preference Index Calculations

The ACL (average chain length) presents the average number of carbon atoms per molecule based on the abundance of the C27, C29, and C31 “higher plant” n-alkanes. This parameter is defined by the following formula: *n*-alkane ACL = (27 × [C27] + 29 × [C29] + 31 × [C31] + … + 37 × [C37])/([C27] + [C29] + [C31] + … + × [C37]), where [Cn is the concentration of the alkane containing × carbon atoms [55].

The CPI (carbon preference index) = (∑odd C from the range of 27 to 37)/(∑even C from the range of 28 to 36) [21].

### 4.5. Bioclimatic Data

The 19 bioclimatic characteristics of all locations of the analyzed populations were extracted from the WorldClim set of global climate layers [56] and Koppen–Geiger climate layer [57]. The bioclimatic parameters were extracted using Q-GIS software (3.22.5). The soil was sampled at three points in each locality, at a depth between 5 and 20 cm. The soil was dried at room temperature and stored in paper bags until further analysis. The pH was measured using ddH_2_O, following the method by Carter and Gregorich [58], with slight modifications. The ratio of ddH20 and soil material was 2.5:1 for predominant mineral soils (instead of 2:1) and 10:1 for predominant organic soils.

### 4.6. Micromorphological Analysis

From all 24 populations collected, the middle leaves were selected for observation. The adaxial and abaxial sides of the leaves were observed and analyzed under a scanning electron microscope (SEM). Additionally, free-hand cross-sections of the dried leaves were carefully positioned on the double-side adhesive tape to allow visibility under the SEM and to measure the thickness of the cuticle. Subsequently, the plant specimens were placed on metal stubs (8 mm diameter) and sputter-coated with gold (BAL-TEC SCD 005). The coating at 30 mA lasted 90 s at a distance of 50 mm. The micromorphological examination was conducted at the University Centre for Electron Microscopy in Novi Sad using a scanning electron microscope JEOL JSM 6460 LV (JEOL, Tokyo, Japan). The micrographs were analyzed using Digimizer software 4.3.5. SEM micrographs were colored using Portable Photoshop CS6. While measuring the thickness of a cuticle by visualization under the SEM is not as robust as using standard light microscopy methods, we opted for this approach for a quick screening of differences in the thickness of the cuticles between the samples. Ten leaves per population were observed under a binocular microscope to determine the density of glandular trichomes expressed as the number of trichomes per mm^2^.

### 4.7. Statistical Analysis

Standard statistics (mean, standard deviation, distribution) were used to study data before univariate (analysis of variance—ANOVA) and multivariate analyses (Principal Component Analysis—PCA; Canonical Discriminant Analysis—CDA; and Multivariate ANOVA—MANOVA). Simple linear correlation and multivariate correlation (Mantel and partial Mantel tests, and Canonical Correspondence Analysis—CDA) were used to analyze the correlation of *n*-alkanes with bioclimatic and orographic data. All statistical analyses were performed using PAST 4.11. [59] on raw data, and only bioclimatic data were log-transformed prior to multivariate analysis (log(x+8)).

## 5. Conclusions

The alkane profile showed the differentiation of the *Acinos*-group from the other taxa based on the dominant *n*-C31, while most of the other taxa contained *n*-C33 as the dominant alkane. However, the alkane profiles differed significantly between species, separating them clearly. A high correlation between glandular trichomes and branched alkanes showed that the *iso*- and *anteiso*-alkanes present in the leaves of *Clinopodium* species suggest that they play a role in enhancing scent release. The micromorphological features also showed clear differences between the previously recognized genera, especially the capitate trichomes. The *Acinos*-group includes as many as nine subtypes of capitate trichomes, the *Calamintha*-group has four subtypes, *Pseudomelissa* has two subtypes, and *C. vulgare* has only one subtype of capitate trichomes. There is no unique indumentum pattern in *Clinopodium,* and the question arises as to which criteria are decisive for including a particular taxon in *Clinopodium*. It could be seen that the micromorphological pattern varies in certain groups of the genus *Clinopodium*. In the *Acinos*-group, interpopulation variability is greater than the variability found between taxa, so they form a very ambiguous complex. In the *Calamintha*-group, the micromorphological patterns are more stable and clearly separate the species. In future research, it would be very useful to study the variations in microclimatic factors in the habitats of the populations. Perhaps this would provide the answer to the question of why the patterns of variability are so pronounced. Moreover, a broad sampling of representatives of the species (belonging to formerly recognized genera *Micromeria*, *Calamintha*, and *Acinos*) is needed to make a more accurate conclusion regarding the usefulness of these parameters as taxonomic markers. Additional morphological and molecular analyses would provide a much clearer understanding of the taxa of this group.

## Figures and Tables

**Table 1:** *Clinopodium* leaf alkane profiles.

	*n*-Alkanes		*iso*-Alkanes		*anteiso*-Alkanes			
	Range ^a^	Total %^b^	Range ^a^	Total %^b^	Range ^a^	Total % ^b^	ACL ^c^	CPI ^d^
AC	21–35	90.4 ± 1.5	29–37	7.1 ± 1.4	30–36	2.5 ± 0.3	31.7 ± 0.2	7.4 ± 1.3
AL	21–35	90.0 ± 1.7	29–37	6.5 ± 1.8	30–36	3.5 ± 0.6	31.8 ± 0.1	9.4 ± 2.3
ALA	21–35	93.0 ± 1.0	30–37	4.5 ± 0.6	30–36	2.5 ± 0.5	32.0 ± 0.1	7.1 ± 1.0
ALH	21–35	88.7 ± 2.5	27–37	7.6 ± 1.6	30–36	3.6 ± 1.3	31.8 ± 0.2	8.2 ± 1.8
ME	21–36	89.9 ± 4.9	27–37	5.6 ± 2.5	30–36	4.5 ± 2.5	32.4 ± 0.3	7.5 ± 2.6
PU	21–35	88.4 ± 1.2	29–37	7.7 ± 1.4	30–36	3.9 ± 0.3	32.1 ± 0.1	10.0 ± 0.2
SU	21–35	90.1 ± 2.8	29–37	5.6 ± 1.2	29–36	4.3 ± 1.8	32.1 ± 0.1	7.1 ± 1.1
TH	21–35	91.7 ± 1.0	29–37	4.5 ± 1.0	29–36	3.8 ± 0.2	32.3 ± 0.1	10.8 ± 2.9
VA	21–35	87.0 ± 2.9	29–37	9.3 ± 2.6	29–36	3.6 ± 1.2	32.2 ± 0.1	6.8 ± 0.5
VU	21–36	94.4 ± 0.8	29–37	2.3 ± 0.8	30–36	3.3 ± 0.6	32.1 ± 0.1	11.4 ± 1.6

^a^ Carbon chain length range, ^b^ calculated as percentage of total area on FID, ^c^ Average chain length, ^d^ carbon preference index. AC—*C. acinos*; AL—*C. alpinum* subsp. *alpinum*; ALA—*C. alpinum* subsp. *albanicum*; ALH—*C. alpinum* subsp*. hungaricum*; ME—*C. menthifolium*; PU—*C. pulegium;*TH—*C. thymifolium*; SU—*C. suaveolens*; VA—C. *vardarense*; VU—C. *vulgare*. For full table, cf. Supplementary Table S1.

**Table 2:** The micromorphological characteristics of examined *Clinopodium* taxa.

Taxa	Cuticle Thickness	Leaf Side	P	C	NG	Density of Peltate Trichomes	Presence of C-Type Trichomes	Presence of NG-Type Trichomes
AC	1.415–3.819	AD	-	+	+	1.89 + 1.1	**C1a**, C2a, C2b, C2c	**NG1**, **NG2**, NG3
		AB	+	+	+	3.22 + 0.70	**C1a**, C1b, C2a, C2b, C2c, C2d	**NG1**, **NG2**, NG3
AL	1.467–3.357	AD	-	+/++	+/++	-	**C1a**	**NG1**, NG2, NG3
		AB	+	+/++	+/++	2.49 ± 0.52	**C1a**, C1b, C2a, C2b, C2c, C2d	**NG1**, NG2, NG3
ALA	1.558–2.570	AD	-	+	++	-	**C1a**	NG1, NG2
		AB	+	+	+	5.38	**C1a**, C1b, C2a	NG1, NG3
ALH	0.885–3.109	AD	-/+	++	+/++	-	**C1a**, C1c, C2a, C2b, C2c, C2d, C2e	**NG1**, NG2, NG3
		AB	+	++	+	2.67 ± 0.69	**C1a**, C1c, C1d, C2a, C2b, C2c, **C2d**	**NG1**, NG2, NG3
SU	0.504–4.077	AD	-/+	+	+/++	3.43 ± 3.97	**C1a**	NG1, NG2, NG3
		AB	+	+/++	+/++	5.91 ± 2.49	**C1a,** C2a, C2b, C2d	**NG1, NG2, NG3**
ME	1.226–5.156	AD	-	+	+	-	**C1a**	**NG1**, NG2, **NG3**
		AB	++	+	+/++	16.52 ± 5.53	**C1a**	NG1, **NG2**, NG3
VA	1.007–4.466	AD	+	++	+/++	3.91 ± 0.72	**C1a**, C2a, C2b, C2c	**NG3**
		AB	++	++	+/++	18.85 ± 1.75	**C1a**, C2a, C2b, C2c	**NG3**
VU	1.427–3.521	AD	-	+	+/++	-	**C1a**	**NG1, NG3**
		AB	+	+	++	3.11 ± 0.94	**C1a**	NG1, **NG3**
PU	2.293–5.019	AD	+	++	++	4.19 ± 1.84	**C1a,** C2c	NG2, NG3
		AB	++	++	++	19.54 ± 4.47	**C1a**, C2c	NG2, NG3
TH	0.846–3.387	AD	+	+	-	6.09 ± 1.77	**C1a**	-
		AB	++	++	+	24.92 ± 7.15	**C1a**	NG1

AD—adaxial leaf side; AB—abaxial leaf side; P—peltate trichomes; C—capitate trichomes; NG—non-glandular trichomes. Presenting the frequency of each trichome type: ‘-’—trichome type absent; ‘+’—trichome type moderately present; ‘++’—trichome type abundant. The density of peltate trichomes is expressed as a trichome number/mm^2^ of leaf surface. The trichome types present in each studied population of certain taxa are in bold. Trichome types not shown in bold were population-specific.

**Table 3:** Details on the localities of the sampled populations.

Taxon	Code	Locality	Longitude [°N]	Latitude [°E]	Alt [m.a.s.l.]	BEOU
*Clinopodium vulgare* L.	VU1	Serbia, Mt. Kosmaj	44.476	20.577	580	18,008
	VU2	Serbia, Tekija	44.632	22.376	488	18,009
	VU3	Serbia, Mt. Rogozna	43.026	20.567	1060	18,010
*Acinos*-group						
*C. acinos* Kuntze	AC1	Serbia, Mt. Tara	43.865	19.406	872	17,987
	AC2	Serbia, Subotica sands	46.139	19.615	133	17,988
	AC3	Serbia, Svrljig gorge	43.542	22.177	259	17,989
*C. alpinum* Kuntze subsp. *alpinum*	AL1	Serbia, Topli Do	43.334	22.664	690	17,990
	AL2	Serbia, Mt. Stolovi	43.607	20.611	1308	17,991
*C. alpinum* subsp. *albanicum* (Kümmerle & Jáv.) Govaerts	ALA1	Serbia, Mt. Rogozna	43.045	20.521	882	17,992
*C. alpinum* subsp. *hungaricum* (Simonk.) Govaerts	ALH1	Serbia, Mt. Fruska gora	45.156	19.778	387	17,993
	ALH2	Serbia, Milesevka gorge	43.367	19.719	604	17,994
	ALH3	Serbia, Gradasnica	43.189	22.597	488	17,995
*C. suaveolens* Kuntze	SU1	Serbia, Soko grad fortress	43.636	21.897	414	18,000
	SU2	Serbia, Mt. Rtanj	43.767	21.926	791	18,001
	SU3	North Macedonia, Raec gorge	41.437	21.878	257	18,002
	SU4	North Macedonia, Dojran	41.197	22.72	166	18,003
*Calamintha* group						
*C. menthifolium*(Host) Stace	ME1	Serbia, Zlot	44.029	21.961	303	17,996
	ME2	Serbia, Mt. Rogozna	43.026	20.567	1060	17,997
	ME3	Serbia, Milesevka gorge	43.367	19.719	604	17,998
*C. vardarense* (Šilić) Govaerts	VA1	North Macedonia, Kaj-Baba	41.665	20.605	796	18,005
	VA2	North Macedonia, Dojran	41.197	22.72	166	18,006
	VA3	Serbia, Dag Banjica	43.203	22.606	499	18,007
*Pseudomellisa* group						
*C. pulegium* (Rochel) Bräuchler	PU1	Serbia, Svrljig gorge	43.543	22.178	259	17,999
*C. thymifolium* Kuntze	TH1	Serbia, Mt. Tara	43.866	19.407	872	18,004

## Data Availability

Data are contained within the article and Supplementary Materials.

## Supplementary Materials

The following supporting information can be downloaded at https://www.mdpi.com/article/10.3390/plants13020251/s1: Figure S1: SEM micrographs of all observed populations from *Acinos*-group; Figure S2: SEM micrographs of all observed populations from *Calamintha*-group, *Pseudomellisa*-group, and *C. vulgare*; Figure S3: PCA scatter plot of bioclimatic parameters of studied localities; Figure S4: PCA scatter plot of alkane profiles of sympatric populations; Figure S5: Soil pH values for studied *Clinopodium* taxa; Table S1: Chemical composition and ANOVA of alkane profiles of studied *Clinopodium* L. taxa from the Balkans; Table S2: Descriptions of each trichome type in *Clinopodium* species.
