# Supplementary material for: Clinopodium L. Taxa from the Balkans—Are There Unique Leaf Micromorphological and Phytochemical Patterns?
Source: Plants (Basel). 2024 Jan 16;13(2):251. doi: 10.3390/plants13020251 (PMC10819394; doi:10.3390/plants13020251)
Supplement: Supplementary file 1 [file plants-13-00251-s001.zip › plants-2793710-2nd xml-supplementary.pdf]

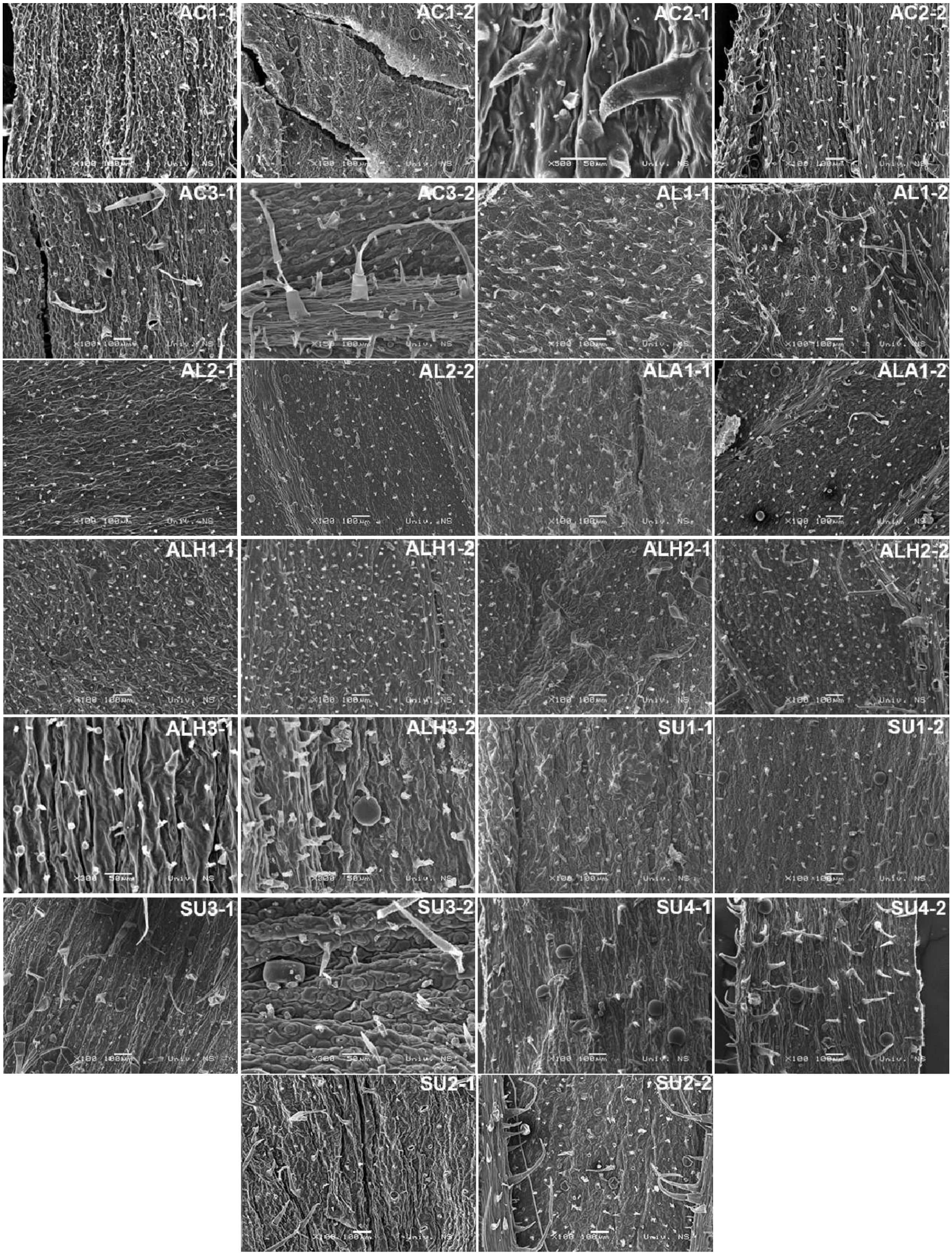

Figure S1. SEM micrographs of all observed populations from *Acinos*-group. For populations' details cf. Table 3; Adaxial (1) and abaxial leaf side (2). AC - *C. acinos*; AL - *C. alpinum*; ALA - *C. alpinum* subsp. *albanicum*; ALH - *C. alpinum* subsp. *hungaricum*; SU - *C. suaveolens*.

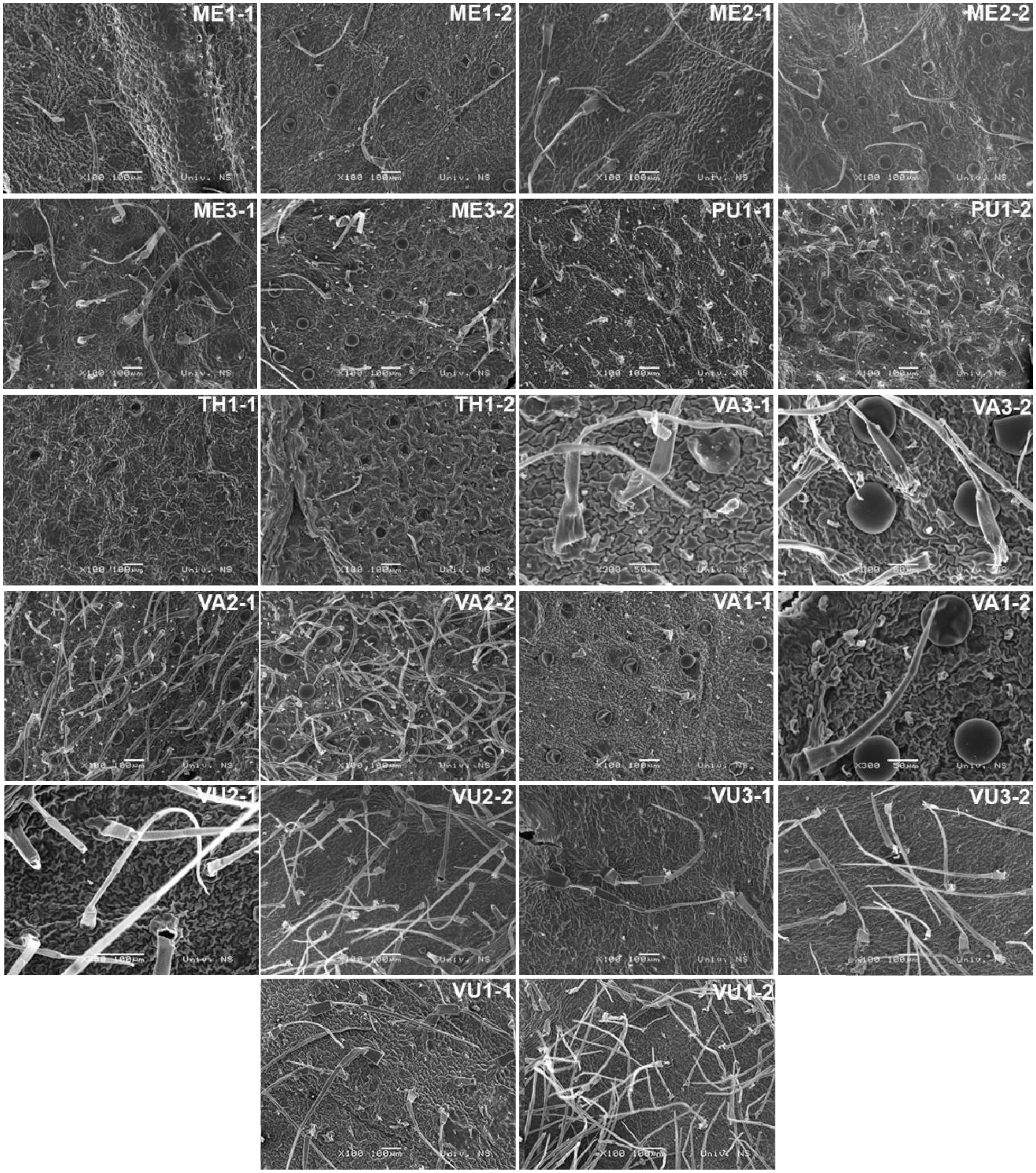

Figure S2. SEM micrographs of all observed populations from *Calamintha*-, *Pseudomellisa*- groups and *C. vulgare*. For populations' details cf. Table 3; Adaxial (1) and abaxial leaf side (2). ME - *C. menthifolium*; PU - *C. pulegium*; TH - *C. thymifolium*; VA - *C. vardarense*; VU - *C. vulgare*.

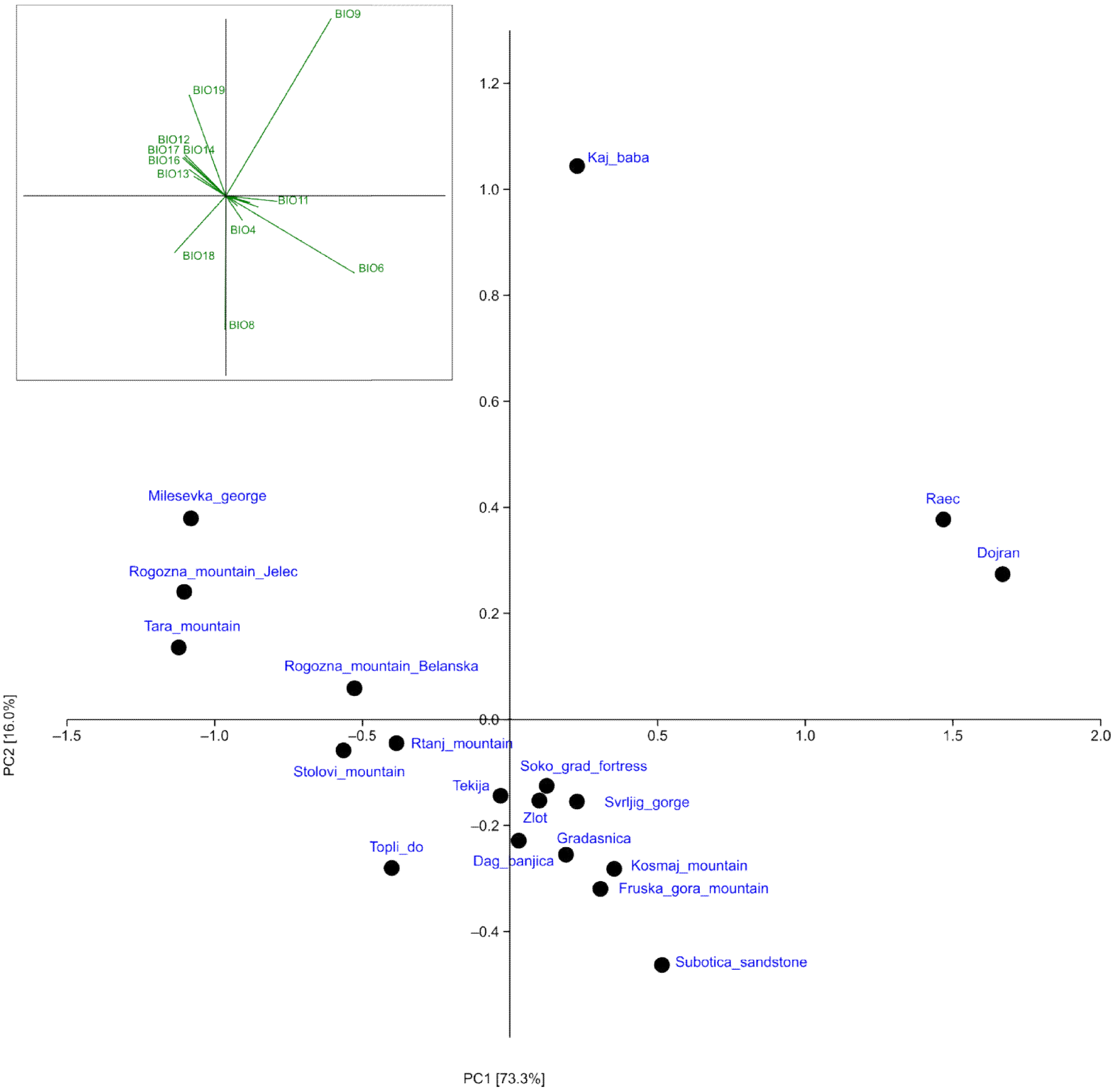

Figure S3. PCA scatter plot of bioclimatic paramteres of studied localities. BIO6 - mean temperature of the coldest month, BIO8 - mean temperature of the wettest quarter, BIO9 – mean temperature of the driest quarter; BIO12 - annual precipitation; BIO18 - precipitation of the warmest quarter, BIO19 - precipitation of the coldest quarter.

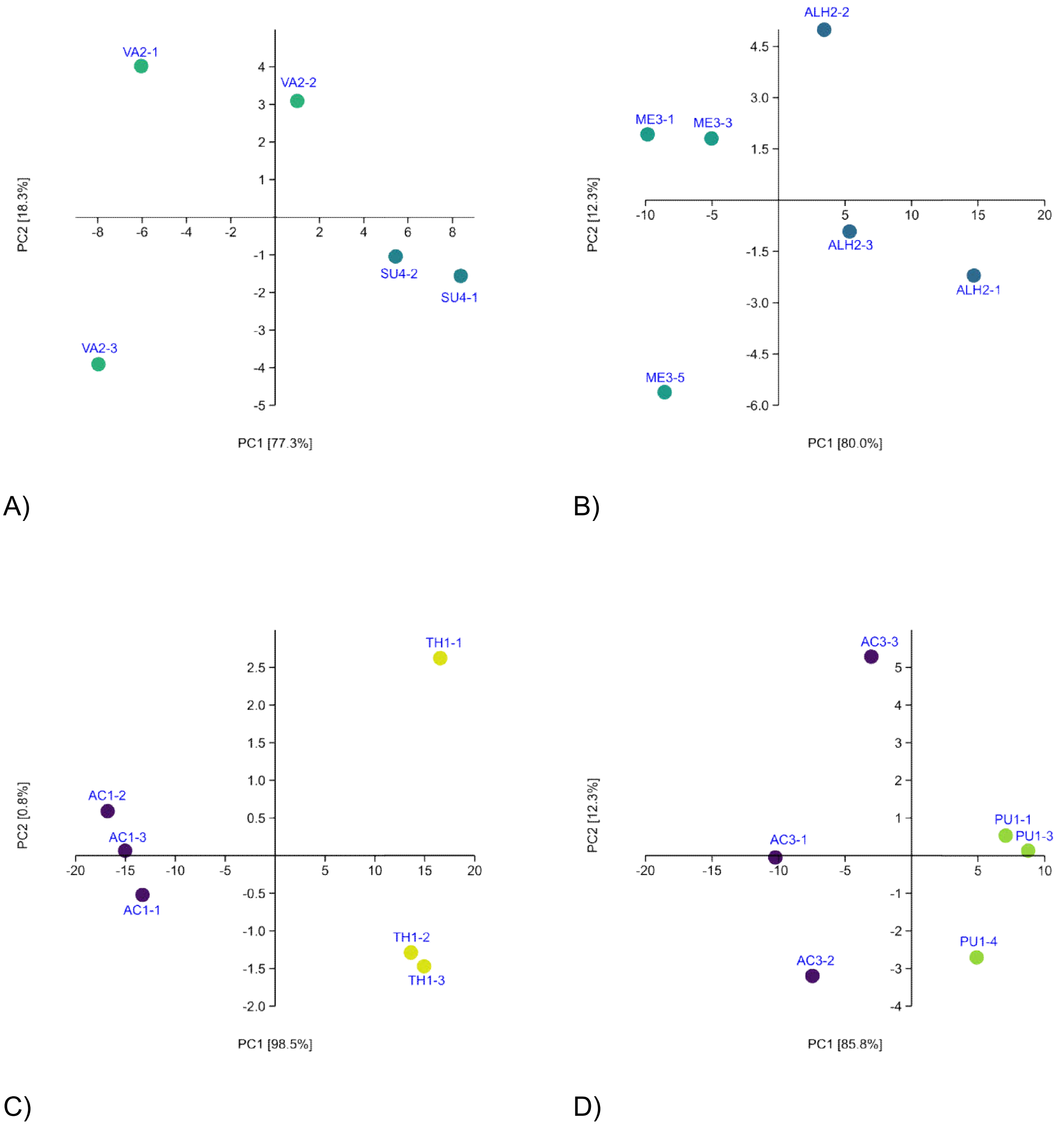

Figure S4. PCA scatter plots of alkane profiles of sympatric populations. A) Dojran, VA - *Clinopodium vardarense*, SU - *C. suaveolens*; B) Milesevka gorge, ME - *C. menthifolium*, ALH - *C. Alpinum* subsp. *hungaricum*; C) Mt. Tara, AC - *C. acinos*, TH - *C. thymifolium*; D) Podvis, AC - *C. acinos*, PU - *C. pulegium.*

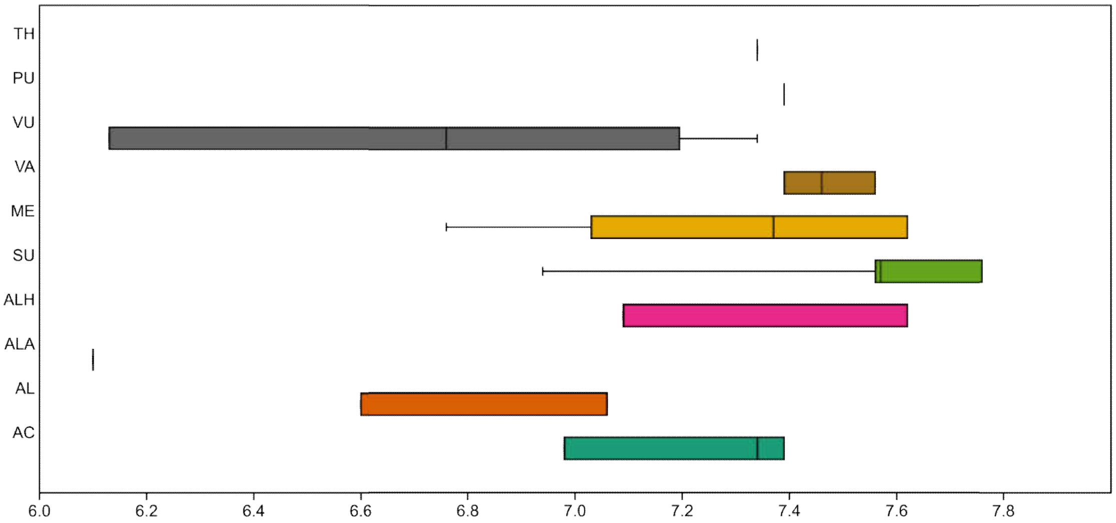

Figure S5. Soil pH values for studied *Clinopodium* taxa: AC - *C. acinos*, AL - *C. alpinum*, ALA - *C. alpinum* subsp. *albanicum*, ALH - *C. alpinum* subsp. *hungaricum*, ME - *C. menthifolium* , PU - *C. pulegium* , TH - *C. thymifolium*, SU - *C. suaveolens* , VA - C. *vardarense* , VU - C. *vulgare.*

Supplementary table S1. Chemical composition and ANOVA of alkane profiles of studied *Clinopodium* L. taxa from the Balkans

		AC	AL	ALA	ALH	ME	PU	SU	TH	VA	VU
	LRI					*n*- alkanes					
C21	2100	tr	tr	tr	tr	tr	tr	tr	tr	tr	tr
C22	2200	0.3±0.17	0.2±0.15	tr	tr	0.2±0.14	tr	0.2±0.06	tr	tr	0.2±0.08
C23	2300	tr	tr	tr	tr	0.2±0.1	tr	0.2±0.07	0.2±0.06	tr	tr
C24	2400	0.3±0.07	0.2±0.06	tr	tr	0.2±0.18	tr	0.3±0.1	0.2±0.01	tr	0.2±0.05
C25	2500	0.4±0.12	0.3±0.06	0.2±0.02	0.3±0.13	0.4±0.3	0.2±0.03	0.5±0.21	0.4±0.03	0.2±0.05	0.3±0.07
C26	2600	0.7±0.2	0.5±0.16	0.3±0.06	0.4±0.57	0.6±0.37	0.2±0.01	0.6±0.2	0.5±0.08	0.3±0.13	0.4±0.14
C27	2700	1.7±0.56	1.9±0.78	0.6±0.01	1.6±1.15	1.6±0.74	1.1±0.32	1.6±0.59	2.1±0.93	1.2±0.19	1.1±0.18
C28	2800	0.8±0.16	0.6±0.18	0.4±0.02	0.5±0.24	0.8±0.54	0.4±0.11	0.8±0.33	0.5±0.17	0.6±0.17	0.5±0.12
C29	2900	8.6±2.57	8.6±1.23	5.1±1.29	7.7±2.74	6.5±2.59	5.4±0.49	5.8±1.53	5.4±1.19	6.0±2.37	8.3±1.11
C30	3000	3.4±0.65	2.2±0.86	2.0±0.51	2.2±0.86	1.7±0.65	1.5±0.32	2.4±0.94	1.2±0.34	1.9±0.56	1.1±0.21
C31	3100	**31.8±2.67**	**31.4±4.6**	**34.3±4.7**	**31.1±4.24**	15.3±4.56	**26.3±1.92**	**25.2±3.52**	16.6±1.54	19±2.14	**24.7±3.37**
C32	3200	8.3±1.66	6.5±1.67	7.2±1.59	7.1±0.95	5.1±1.25	4.1±0.69	7.1±0.92	5.9±1.24	6.5±0.51	4.1±0.44
C33	3300	**29.7±4.35**	**34.3±3.86**	**39.4±4.16**	**34.3±3.5**	**44.6±9.78**	**46.4±1.3**	**40.4±5.37**	**51.0±1.16**	**44±3.88**	**50.0±4.00**
C34	3400	1.9±0.37	1.5±0.11	1.8±0.12	1.6±0.37	5.1±1.09	1.3±0.13	2.7±0.55	1.6±0.21	3.9±0.54	0.9±0.59
C35	3500	2.0±0.89	1.1±0.12	1.3±0.4	1.1±0.43	7±4.59	0.9±0.21	1.9±0.85	5.5±1.3	2.7±0.73	2.4±1.15
C36	3600	-	-	-	-	tr	-	-	-	-	tr
Total		90.4±1.54	90.0±1.72	93.0±0.98	88.7±2.53	89.9±4.92	88.4±1.17	90.1±2.78	91.7±1.01	87.0±2.9	94.4±0.8
						iso- alkanes					
C27	2659	-	-	-	tr	tr	-	-	-	-	-
C28	2759	-	-	-	tr	tr	-	-	-	-	-
C29	2859	tr	tr	-	0.2±0.25	tr	tr	tr	tr	0.9±2.31	tr
C30	2959	tr	tr	tr	tr	tr	tr	tr	tr	tr	tr
C31	3059	1.6±0.44	1.5±0.69	0.6±0.09	1.5±0.43	0.7±0.39	2.1±0.89	1.0±0.27	1.0±0.1	1.2±0.69	0.3±0.07
C32	3159	0.3±0.09	0.4±0.16	0.3±0.08	0.5±0.17	0.2±0.13	0.4±0.11	0.4±0.13	0.4±0.07	0.4±0.16	tr
C33	3259	**4.0±1.05**	**3.7±1.06**	**2.8±0.37**	**4.7±1.42**	**2.8±1.38**	**4.5±1.2**	**3.4±0.99**	**2.7±0.7**	**5.3±1.38**	**1.0±0.1**
C34	3359	0.4±0.34	0.4±0.12	0.4±0.07	0.4±0.12	0.3±0.14	0.4±0.06	0.5±0.2	0.4±0.05	0.5±0.18	0.7±0.95
C35	3459	0.9±0.13	0.7±0.08	0.6±0.13	0.7±0.12	1.4±0.63	0.4±0.17	0.5±0.39	0.3±0.1	1.2±0.35	tr
C36	3559	-	-	-	tr	tr	-	-	-	-	tr
C37	3659	tr	tr	tr	tr	tr	-	tr	-	0.2±0.36	-
Total		7.1±1.38	6.5±1.79	4.5±0.6	7.6±1.61	5.6±2.48	7.7±1.4	5.6±1.15	4.5±0.99	9.3±2.55	2.3±0.79
						anteiso- alkanes					
C29	2871	-	-	-	-	-	-	tr	tr	tr	-
C30	2971	tr	tr	tr	tr	tr	tr	tr	0.2±0.05	0.3±0.69	tr
C31	3071	-	-	tr	tr	tr	tr	tr	tr	tr	tr
C32	3171	0.8±0.24	0.9±0.31	0.7±0.16	1.0±0.38	0.6±0.37	0.8±0.16	0.9±0.39	1.3±0.11	0.7±0.24	0.7±0.16
C33	3271	0.2±0.06	0.3±0.10	0.2±0.02	0.3±0.09	0.1±0.11	0.3±0.11	0.4±0.27	0.4±0.02	0.2±0.05	0.1±0.04
C34	3371	**1.1±0.44**	**1.5±0.31**	**1.2±0.3**	**1.8±0.71**	**2.6±1.50**	**2.3±0.32**	**1.9±0.71**	**1.5±0.31**	**2.0±0.97**	**2.0±0.56**
C35	3471	tr	0.2±0.07	tr	tr	0.2±0.1	tr	tr	tr	tr	tr
C36	3571	0.3±0.09	0.4±0.07	0.3±0.02	0.3±0.14	0.9±0.46	0.2±0.02	0.7±0.57	0.2±0.02	0.2±0.1	0.2±0.11
Total		2.5±0.33	3.5±0.63	2.5±0.52	3.6±1.27	4.5±2.54	3.9±0.25	4.3±1.8	3.8±0.23	3.6±1.18	3.3±0.63
ACL		31.7±0.19	31.8±0.07	32.0±0.04	31.8±0.19	32.4±0.32	32.1±0.07	32.1±0.13	32.3±0.09	32.2±0.1	32.1±0.13
CPI		7.4±1.3	9.4±2.32	7.1±0.98	8.2±1.82	7.5±2.63	10.0±0.23	7.1±1.14	10.8±2.9	6.8±0.51	11.4±1.59

		groups according to Tukey's post-hoc test									
		AC	AL	ALA	ALH	ME	PU	SU	TH	VA	VU
	p						*n*- alkanes				
C21	-	a	a	a	a	a	a	a	a	a	a
C22	***	a	a,b	b	b	a,b	b	a,b	a,b	b	a,b
C23	***	a,b	a,b	a,b	a,b	a,b	b	a	a,b	b	a,b
C24	***	a	a,b	a,b	b	a,b	b	a	a,b	b	a,b
C25	***	a,b	a,b	a,b	a,b	a,b	a,b	a	a,b	b	a,b
C26	***	a	a	a	a	a	a	a	a	a	a
C27	***	a	a	a	a	a	a	a	a	a	a
C28	***	a	a	a	a	a	a	a	a	a	a
C29	***	a	a	a	a	a	a	a	a	a	a
C30	***	a,b,c	a,b,c,d	a,b,c,d	a,b,c	b,c,d	a,b	a,b,c,d	a,b,c	a,b,c,d	c,d
C31	***	a	a	a	a	b	ac	ac	b	bc	c
C32	***	a,f,g,h,i	a,h	a,h,i	a,f,h,i	a,b,e	a,b,d,e	a,h,i	a	a,b,h	a,f,h,i
C33	***	a,b	a,c	a	a,c	a,d	a,d	a,e	a,d	a,d	a,f
C34	***	a	a	a	a	a	a	a	a	a	a
C35	***	a,b	a	a,b	a	c	a	a,b	d	a,b	a,b
C36	-	a	a	a	a	a	a	a	a	a	a
Total	***										
	p						*iso*- alkanes				
C27	-	a	a	a	a	a	a	a	a	a	a
C28	-	a	a	a	a	a	a	a	a	a	a
C29	-	a	a	a	a	a	a	a	a	a	a
C30	***	a	a,b	a,b	a,b	a,b	a,b	a,b	a,b	b	a,b
C31	***	a,c	a,c	a	a,c	b	a,c	c	b	b	c
C32	***	a	a	a,b	a,c	a,b	a	a	a	a	b
C33	***	a,b	a,b	a	a,b	a,c	a,d	a,b	a,c	a,e	a,f
C34	***	a	a	a	a	a	a	a	a	a	a
C35	***	a,c	a,d	a,b,d	a,e	c	a,b	a,b	a,b	a,c	b
C36	***	a	a	a	a	a	a	a	a	a	a
C37	-	a	a	a	a	a	a	a	a	a	a
Total	***										
	p						*anteiso*- alkanes				
C29	-	a	a	a	a	a	a	a	a	a	a
C30	***	a,c	a,b,c	a,b,c	b,c	b,c	b,c	a,c	b,c	b,c	b
C31	-	a	a	a	a	a	a	a	a	a	a
C32	***	a	a	a	a	a,b	a	a	a,c	a	a
C33	***	a,b	a	a	a	a,b	a	c	a	a,b	a,b
C34	***	a	a	a	a	b	a	a	a	a	a
C35	***	a,b	a,c	a	a	a,c	a	a	a	a	a,b
C36	***	a,b	a	a	a,b	a,c	a,b	a,d	a,b	a,e	a,e
Total	***	a	a	a	a	a	a	a	a	a	a
	p										
ACL	***	a,b,c	a,b,c,d	a,b,c,d,f	a,b,c,d	e,f	a,b,c,d,e,f	b,c,d,f	c,e,f	c,e,f	b,c,d,f
CPI	***	a,c	a,b,c	a,c	a,c	a,c	a,c	a,c	a,c	a	b,c

Coulmns with the same letter do not differ significantly according to the Tukey's post-hoc test; p: statistical significance of ANOVA -: $p>0.05$; *** $p<0.01$

AC - C. *acinos* , AL - C. *alpinum* , ALA - C. *alpinum* subsp. *albanicum* , ALH - C. *alpinum* subsp. *hungaricum* , ME - C. *menthifolium* , PU - C. *pulegium* , TH - C. *thymifolium* , SU - C. *suaveolens* , VA - C. *vardarense* , VU - C. *vulgare*

Supplementary table S2. Descriptions of each trichome type in *Clinopodium* species

Designation	Description of certain trichome type
Peltate trichomes - **P**	They consisted of a short stalk deeply embedded in the epidermis and a broad, multicellular head with a large subcuticular space.
Capitate trichome - subtype **C1a**	Consists of a very short stalk cell (up to 10 μm) and an elliptical to obovoid-shaped head cell that is larger than the stalk (from 10-20 μm). In the post-secretory phase, the very thin cuticle of the head and stalk becomes invaginated, giving these trichomes the appearance of various irregular formations adhering to epidermal cells.
Capitate trichome - subtype **C1b**	The stalk cell is rigid, non-ruptured in the post-secretory phase, trapezoidal with longitudinal cuticular folds and tapers towards the head. The head is round and has a diameter of 10-15 μm.
Capitate trichome - subtype **C1c**	The head and the stalk of approximately equal in diameter (about 25 μm). The stalk cell is rigid and trapezoidal, while the head is obovoid.
Capitate trichome - subtype **C1d**	Consists of a rigid, papillose, trapezoidal stalk cell, a thin neck and a globular head. The main characteristic which distinguished this type from the others is the presence of the neck cell with a diameter of about 20 μm.
Capitate trichome - subtype **C2a**	A stalk (25-30 μm) consisting of two cells of approximately equal size, but the lower cell is more rigid and has characteristic longitudinal folds, while the upper cell has clear invaginations of the cuticle. The head is globular, 10-15 μm in diameter.
Capitate trichome - subtype **C2b**	Consists of a globular to obovoid head (15-25 μm) and two-celled stalk (30-40 μm). The lower cell of the stalk is predominantly higher, rigid and trapezoidal, while the upper cell is irregular, short and has a thinner cuticle.
Capitate trichome - subtype **C2c**	The stalk is 15-20 μm high, with noticeably shorter and more rigid lower cell. Two cells of the stalk have the same weight and the head is broader, globular, and has a diameter of about 15 μm.
Capitate trichome - subtype **C2d**	This subtype has a stalk (approx. 40 μm) consisting of two cells with the same height but different shapes. The lower cell is somewhat thinner and more rigid, while the upper cell widens trapezoidally towards the head. These two cells form a characteristic knee-like extension between them.
Capitate trichome - subtype **C2e**	The subtype has a short stalk (20 μm) consisting of two rigid cells with a clear triangular shape and wide head.
Non-glandular trichomes - subtype **NG1**	The unicellular trichomes are short, triangular, mostly arranged upright or diagonally to the epidermis and the smallest of all trichomes in this group.
Non-glandular trichomes - subtype **NG2**	The bicellular trichomes consist of the lower cell which is usually rigid and trapezoidal, and the narrower upper.
Non-glandular trichomes - subtype **NG3**	The third group includes the long trichomes, which consist of three or more cells, in multicellular trichomes, the plane of one cell is rotated over the others.
